# Diagnosis of apical hypertrophic cardiomyopathy: T-wave inversion and relative but not absolute apical left ventricular hypertrophy^☆^

**DOI:** 10.1016/j.ijcard.2015.01.054

**Published:** 2015-03-15

**Authors:** Andrew S. Flett, Viviana Maestrini, Don Milliken, Mariana Fontana, Thomas A. Treibel, Rami Harb, Daniel M. Sado, Giovanni Quarta, Anna Herrey, James Sneddon, Perry Elliott, William McKenna, James C. Moon

**Affiliations:** a Department of Cardiology, University Hospital Southampton, Tremona Road, Southampton SO166YD, United Kingdom; b The Heart Hospital, part of University College London Hospitals NHS Trust, 16-18 Westmoreland Street, London W1G 8P, United Kingdom; c Institute of Cardiovascular Science, University College London, United Kingdom; d Department of Cardiology, S Andrea Hospital, Universtiy Sapienza Rome, Italy; e East Surrey Hospital, Canada Avenue, Redhill RH1 5RH, United Kingdom; f Cardiovascular Department, AO Papa Giovanni XXIII, Bergamo, Italy

**Keywords:** Hypertrophic cardiomyopathy, Cardiovascular magnetic resonance

## Abstract

**Background:**

Diagnosis of apical HCM utilizes conventional wall thickness criteria. The normal left ventricular wall thins towards the apex such that normal values are lower in the apical versus the basal segments. The impact of this on the diagnosis of apical hypertrophic cardiomyopathy has not been evaluated.

**Methods:**

We performed a retrospective review of 2662 consecutive CMR referrals, of which 75 patients were identified in whom there was abnormal T-wave inversion on ECG and a clinical suspicion of hypertrophic cardiomyopathy. These were retrospectively analyzed for imaging features consistent with cardiomyopathy, specifically: relative apical hypertrophy, left atrial dilatation, scar, apical cavity obliteration or apical aneurysm. For comparison, the same evaluation was performed in 60 healthy volunteers and 50 hypertensive patients.

**Results:**

Of the 75 patients, 48 met conventional HCM diagnostic criteria and went on to act as another comparator group. Twenty-seven did not meet criteria for HCM and of these 5 had no relative apical hypertrophy and were not analyzed further. The remaining 22 patients had relative apical thickening with an apical:basal wall thickness ratio > 1 and a higher prevalence of features consistent with a cardiomyopathy than in the control groups with 54% having 2 or more of the 4 features. No individual in the healthy volunteer group had more than one feature and no hypertension patient had more than 2.

**Conclusion:**

A cohort of individuals exist with T wave inversion, relative apical hypertrophy and additional imaging features of HCM suggesting an apical HCM phenotype not captured by existing diagnostic criteria.

## Highlights

• The normal left ventricular myocardium tapers towards the apex.
• There exists an apical HCM variant with relative rather than absolute apical hypertrophy.
• T-wave inversion and relative apical hypertrophy are core features.
• Additional features include a dilated left atrium, scarring, apical cavity obliteration and apical microaneurysms.

## 1 What is already known about this subject?

There are a significant number of patients where conventional investigation with ECG and echocardiography fails to reach a diagnosis but where CMR can diagnose hypertrophic cardiomyopathy. This occurs most frequently in a subtype: apical hypertrophic cardiomyopathy — where these patients are known to be at equal risk from the underlying disease. Despite this, there are many patients where conventional criteria fail to make a diagnosis despite clinical suspicion.

## 2 What does this study add?

This study implies that a prephenotypic variant of HCM exists with extensive T wave inversion on ECG and relative but not absolute apical hypertrophy. These features are best seen using cardiovascular magnetic resonance imaging which can identify patients with disease features who could otherwise be labeled as normal but who may share their fate with patients who have overt HCM. Further study of these patients is needed to track associated morbidity and prognosis.

## 3 How might this impact on clinical practice?

We have identified a group of patients with a distinct phenotype not captured by existing disease classifications. These share the characteristics of patients with classical apical hypertrophic cardiomyopathy (HCM). With the criteria outlined in this article, patients can be identified, followed up and appropriately considered for family screening.

## 4 Introduction

**Fig. 1:**
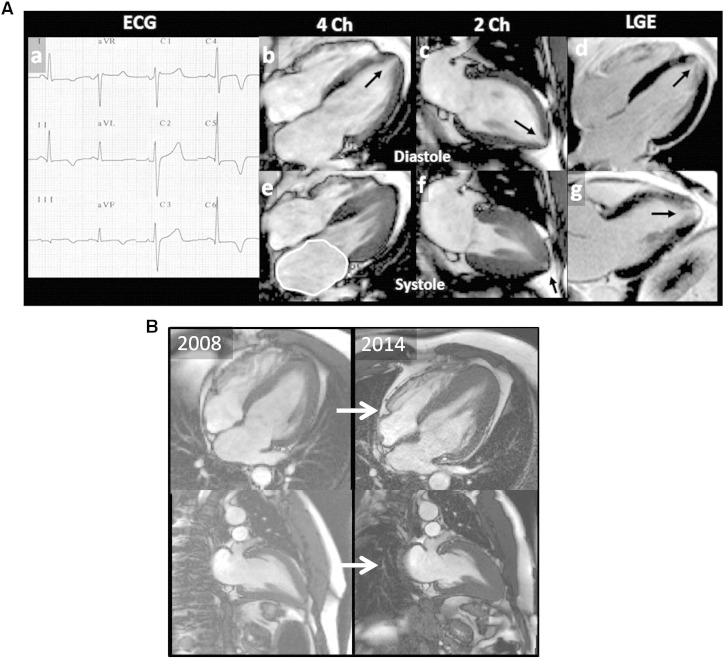
a, Case in point 1 (see online supplementary cine images). An athletic 49 year old man with no family history and normal coronary arteries was referred with atypical chest pain, an abnormal ECG (deep infero-lateral TWI, a) and a normal echocardiogram for CMR. This demonstrated: 1) a discrete increase in wall thickness at the apex (10 mm vs 8 mm basally); 2) a 14 mm tube-like apical cavity (arrowed, b, c) which obliterates in systole; 3) a small apical micro-aneurysm un-obliterated in systole (arrowed, f); 4) apical scar (arrows d, g). 5) Left atrial dilatation (e). This patient was subsequently found to have a gene mutation in myosin binding protein C3 (R810H). b, Case in point 2. Despite the different appearances between the left and right images; all images are in diastole (SSFP cine stills). Top row 4 chamber, bottom row 2 chamber. Left: Index scan 2008 of 47 year old male referred with TWI and atypical chest pain which was related to gall stone disease. Maximum wall thickness in 2008 was 12 mm with apical cavity obliteration of 25 mm. At follow-up 6 years later, the maximum wall thickness was 18 mm with apical cavity obliteration of 41 mm. He now has a formal diagnosis of HCM.

There are patients who present with ECG changes and symptoms that suggest hypertrophic cardiomyopathy (HCM), yet findings on echocardiography are non-diagnostic. Some of these patients are identified using cardiovascular magnetic resonance (CMR) as having HCM but with hypertrophy confined to the apex [1,2]. With the increasing use of CMR to accurately define cardiac anatomy in HCM [3–5], we have noted another group of patients with the same ECG changes but where HCM diagnostic criteria [6–8] (left ventricular wall thickness over 15 mm) are not fulfilled and yet the apex is abnormal with relative rather than absolute apical hypertrophy. These patients often have other imaging abnormalities more commonly observed in HCM patients such as a dilated left atrium, systolic apical cavity obliteration, scar identified on CMR, and apical microaneurysm. See cases in point, Fig. 1.

We hypothesized that individuals with T-wave inversion (TWI) would share similar disease characteristics as seen in HCM and sought to define these characteristics compared to health, established HCM and a key possible differential diagnosis, cardiac changes in hypertension.

## 5 Methods

### 5.1 Patient population

An ethics committee of the UK National Research Ethics Service approved generic, retrospective analysis of anonymized clinical scans. All research was carried out at University College London Hospital NHS Trust, London between October 2008 and June 2010, where a tertiary referral center for both cardiomyopathy and CMR exists. A retrospective analysis of CMR scans and referrals within this period (n = 2662) was performed. We identified 75 patients referred with unexplained T-Wave inversion (TWI) and clinical suspicion of hypertrophic cardiomyopathy. Sixty healthy control volunteers and fifty hypertensive patients were studied as comparator populations. The volunteers were recruited from the hospital staff, the university, a local general practice and a dance class. All underwent a cardiac history, ECG, blood pressure measurement and contrast CMR with no abnormalities detected. Family history of HCM (first degree relative) was obtained from the clinical notes and by telephone interview at the time of analysis. The hypertensive controls (consecutive consenting hypertension patients from a dedicated tertiary care hypertension service with clinic BP > 140/90) underwent ECG and contrast CMR.

Standard 12 lead ECG was obtained in all patients and volunteers The ECG was analyzed for the presence, location, depth and distribution of T-wave inversion in the anterior leads. The presence of T-wave inversion was defined when it extended beyond lead V2, deep TWI was defined as greater than or equal to 5 mm.

### 5.2 CMR acquisition and analysis

Standard CMR examination was performed in all patients [9] on a 1.5 T scanner (Avanto, Siemens, Erlangen, Germany). Appropriate methods were used to optimize image quality. Where conventional long axis views suggested apical abnormalities, additional 5 mm (rather than conventional 7 mm) slices and cross cuts were made (as per our standard practice). For scar imaging, contrast (0.1 mmol/kg, Dotarem — Guerbet, S.A.) was administrated intravenously and standard breath-hold, inversion recovery imaging was performed. Additional confirmatory views for scar were performed where necessary (phase swaps, SSFP read-out, imaging in systole) [9–11]. Myocardial volumes and mass analyses were carried out using standard techniques [9].

### 5.3 Definitions

**Fig. 2:**
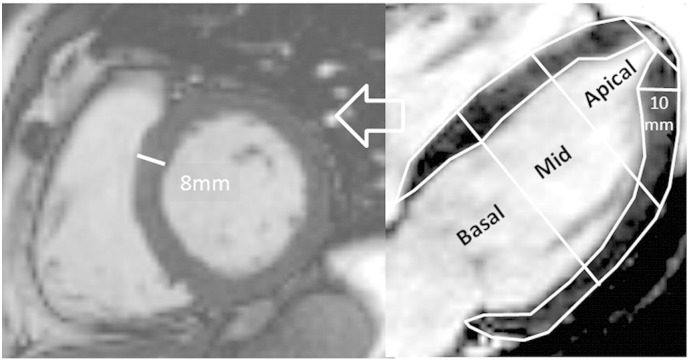
Measuring apical wall thickness. Left: short axis slice near the base of the heart with max wall thickness of 8 mm. Middle: four chamber view. The left ventricle is divided into thirds: Basal, mid and apical, the apical cap makes up only the most apical 6% of the ventricle. At the base the short axis slice is truly perpendicular to the wall. Towards the distal ventricle — as the cavity tapers, the short axis slice is not perpendicular to the wall — this complicates wall thickness measurement and it should be performed on multiple long axis views, perpendicular to the true septal axis and with careful exclusion of papillary muscle origins and trabeculation.

Hypertrophy — apical or elsewhere — was defined as a compacted myocardial wall thickness in diastole greater than or equal to 14 mm, with meticulous exclusion of overlying LV/RV trabeculae (assessed on cine images). Fourteen millimeters (rather than conventional echocardiography thickness of 15 mm) was used as the threshold since CMR measures wall thickness thinner than echocardiography in the basal septal segments [5]. For apical hypertrophy where measurement is potentially fraught, extra care and attention to detail was used. Measurement was performed on the three long axis views using the 7 and/or 5 mm crosscuts and where the imaging plane was not foreshortening the ventricle, visualizing the true cardiac apex (but not the apical cap). In choosing where to measure (septal, anterior, inferior or lateral) the short axis views were consulted and the base of papillary muscles and trabeculae were carefully avoided. Measurement was performed perpendicular to the axis of the wall and at the point of maximal thickness (see Fig. 2).

Relative apical hypertrophy was defined as the absence of hypertrophy (wall thickness < 14 mm) but with the apical wall thickness greater than the basal wall thickness (apex:base ratio (ABR) wall thickness > 1).

Additional imaging disease features of HCM were defined as follows:

1. Systolic anterior motion of the mitral valve ascertained using frame by frame analysis in the three chamber view and or supporting basal short axis view(s).
2. Mitral annular planar systolic excursion (MAPSE) measured using standard techniques and defined for this analysis as abnormal when < 10 mm.
3. Left atrial dilatation, defined in the 4 chamber view with an upper limit of 25 cm^2^ [12].
4. Abnormal systolic apical cavity obliteration was graded as > 20 mm or > 10 mm. Measured in the 5 mm long axis cine crosscuts in the 3 views (4ch, 2ch, LVOT view) from the apical cap to the blood cavity in systole. The minimum of the three measurements was used.
5. An apical aneurysm was defined as persistence of apical blood pool distal to cavity obliteration in systole.
6. Scar by LGE was defined visually by 2 independent observers and defined as present or absent.

### 5.4 Statistics

Data are expressed as mean +/− standard deviation or median and interquartile range (for normal and non-normally distributed data respectively). Group means comparisons were performed using Student's T-test (two groups) given that the a priori hypothesis was that each patient group would differ from health. Chi square test was used to compare frequency of categorical data. A p < 0.05 was considered statistically significant. SPSS [13] Version 17 was used for analysis. Analyses were performed in a blinded fashion.

## 6 Results

Of the 75 patients coronary angiography had been performed in 35 of which 18 had normal coronary arteries, 15 had minor atheroma, 2 had undergone angioplasty (1 left anterior descending artery and 1 to circumflex) — but without sustained symptomatic/ECG improvement such that alternative diagnoses were sought. Forty had not undergone angiography (of which four had a normal perfusion scan).

Patient and comparator group characteristics are presented in Table 1. All CMR scans were of adequate image quality.

**Fig. 3:**
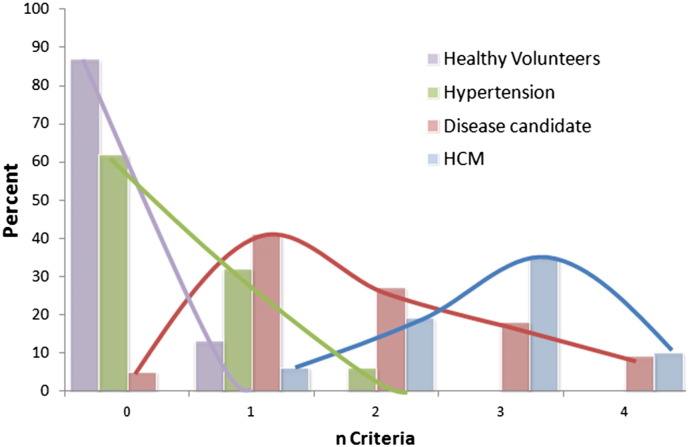
Graph showing number of abnormal criteria fulfilled by disease type. This illustrates that there are many patients in the relative apical hypertrophy group who are much more akin to true HCM than to normal but there is considerable overlap.

Of the 75 patients, 48 met conventional HCM criteria (43 were males with a mean ± SD age of 56 ± 11 years). Of the remaining 27, 22 had relative apical hypertrophy (ABR > 1, 5 other patients with ABR < 1 were not studied further). These 22 patients are the disease candidate group and form the group studied from here on. Of these 22 patients the mean age was 50 ± 15, 9 were females, 18 Caucasian, 3 Black, and 1 Asian. Eight had a documented family history of HCM. One patient had a history of syncope and 5 had non sustained ventricular tachycardia recorded on Holter monitoring. No patient in this group had aortic stenosis or coarctation; 6 patients had a history of mild hypertension (on ≤ 2 antihypertensive agents). Nine had LGE (7 limited to the apex, two had mid-myocardial scar in the area of maximum hypertrophy). They all had TWI (by definition) and 16 (73%) had deep TWI. None of the healthy and hypertensive control groups had TWI. The mean apical wall thickness and ABR was significantly greater in the patients with TWI (1.2 ± 0.1) than in healthy volunteers and hypertensive patients (Table 2 and Fig. 3). No healthy individual had an ABR of > 0.9 and no hypertensive > 0.7 ie. the apical WT was universally thinner than the base. In fact the mean ABR was significantly lower in hypertension than in controls (0.6 ± 0.1 mm vs 0.5 ± 0.1 mm, p < 0.001) even though the mean basal wall thickness was greater (11 ± 0.1 mm vs 8 ± 0.1 mm, p < 0.001). The pattern of LGE in the hypertension group (present in 4 patients) was mid-myocardial and limited to the right ventricular insertion points. Of the 6 additional putative disease features (left atrial dilatation, MAPSE < 10 mm, apical cavity obliteration, apical aneurysm, scar by LGE, or systolic anterior motion of the mitral valve) two were not considered in the analysis: SAM because it occurred in only one case and MAPSE < 10 mm because it was not worse in the candidate disease population compared to hypertension and healthy controls. This left four disease features, all of which occurred at greater prevalence than in the comparator groups. Each had different discriminatory characteristics between the disease candidate group and comparator disease group (see Table 2).

The disease candidate group had at least one disease feature in 95% of cases (compared to 38% of hypertensive's and 18% of healthy controls). The disease candidate group had slightly fewer features than the HCM group but significantly more than the hypertensive and healthy controls (mean: 1.9 vs 2.4, 0.5 and 0.1 and median: 2 vs 2, 0 and 0 respectively, p = 0.001) see Fig. 3. No healthy subject had more than one abnormality present. Only 6% of hypertensive patients had ≥ 2 features compared to 54% of the disease candidate group.

## 7 Discussion

This study describes an unrecognized group of patients who fall outside of conventional diagnostic criteria but have a collection of features suggestive of disease akin to apical HCM. These patients have three characteristics: firstly, characteristic ECG changes with T wave inversion, deep in three quarters. Second, all had relative apical hypertrophy not reaching conventional hypertrophy thickness criteria. Thirdly, there was a prevalence of additional HCM imaging features (LA dilatation, > 2 cm apical cavity obliteration, scar and apical aneurysms) approaching that of classical HCM and beyond that found in healthy controls or hypertension. Our referral base suggests that this cohort is not rare — we have detected 22 such cases in less than two years at our tertiary referral center (specializing in cardiomyopathy and advanced cardiac imaging). Such patients will often receive no definitive diagnosis. They may represent a morphologically mild or a prephenotypic variant of apical HCM. They may share the same predisposition to morbidity as HCM — particularly chest pain, atrial fibrillation, ventricular arrhythmia and eventual progression to overt cardiomyopathy and heart failure. We note that of the 22 patients, 23% had a history of atrial fibrillation.

One other clinical characteristic of the cohort is noted: 4 patients (18%) were veteran athletes (including the case in point, Fig. 1). While this may be referral bias — such individuals may be more subjected to screening and present more to healthcare services. It may be that exercise over many decades, in the predisposed individual, induces myopathic like changes as detected here. Some of the features seen here (left atrial dilatation, borderline hypertrophy) occur in athletes, and TWI in this population may presage overt cardiomyopathy development [14].

The detection of new disease and refinement of existing disease spectra, often occur with technological advances. Apical HCM diagnostic criteria have evolved according to the technology of the time: Initially, diagnosis was made with ECG (deep TWI) [15,16] associated with the spade like cavity obliteration described with angiography [17]. With echocardiography came description of apical hypertrophy [1,18]. Subsequently, CMR identified apical hypertrophy missed by echocardiography in the context of characteristic ECG changes [1,2,19,20]. Additional features, such as apical scar and the prognostic significance of apical aneurysms have recently been described [21]. In this study, the technological driver is CMR but more related to its increased clinical use; reflecting the increased awareness of physicians of missed apical HCM with TWI. Only half of gene carriers have an abnormal echocardiogram or ECG [22] suggesting that incomplete disease expression may be very common. The question is whether the individuals in this cohort represent incomplete disease expression by current criteria or are an extreme of normality. Our data support the former hypothesis and suggest that the current HCM diagnostic criteria should be modified.

**Fig. 4:**
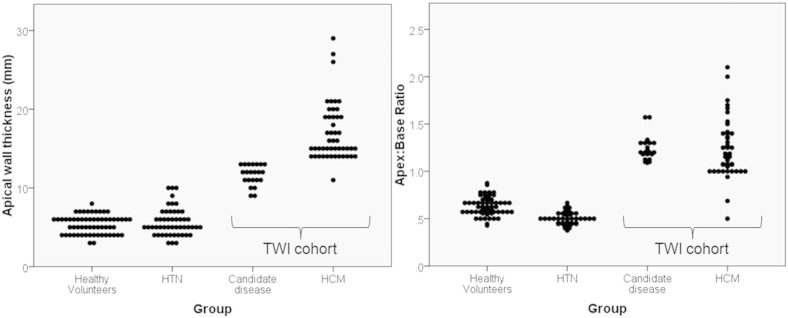
a) Left ventricular apical wall thickness across the groups. In healthy volunteers and hypertension, the apex is a thin structure (mean ± SD = 5 ± 1 and 6 ± 2). In the patients with TWI, the apex is significantly thicker (15 ± 4, p < 0.001 ANOVA). b) In healthy volunteers and hypertension wall thickness universally tapers towards the apex and thus the apex:base wall thickness ratio is always less than 0.9 (mean ± SD apex:base ratio = 0.6 ± 0.1 and 0.5 ± 0.1) unlike the disease candidate group (1.2 ± 0.3, p < 0.001).

A strength of this study is the two comparator groups — healthy volunteers (n = 60) and hypertensive patients (n = 50). In these, no individual had ABR > 0.9. Following Laplace's law as the radius of the ventricle gets smaller towards the apex, as does the wall stress — the natural tendency is for WT to thin towards the apex even when the basal wall has hypertrophied (see Fig. 4). In hypertension for instance, the basal WT was greater than normals but the ABR was less reflecting that hypertrophy in this condition has a basal preponderance. None of the hypertension group had TWI. Based on these two criteria alone (ABR > 1 and TWI), our candidate disease group is entirely distinct but the additional imaging features add certainty: both to the classification as a disease and in diagnostic power. The presence of > 20 mm of apical obliteration was highly discriminatory with no control individual demonstrating this feature. Left atrial dilatation however was common and not specific. Apical aneurysm was an uncommon finding in patients but was not present in controls. The presence of LGE was much higher in the candidate disease group (41%) but this was also present in 8% of hypertensive subjects. The pattern of scar was different — 7 out of the 9 candidate disease group had apical scar. The hypertensive patients all had right ventricular insertion point scar in keeping with previous studies [23].

**Fig. 5:**
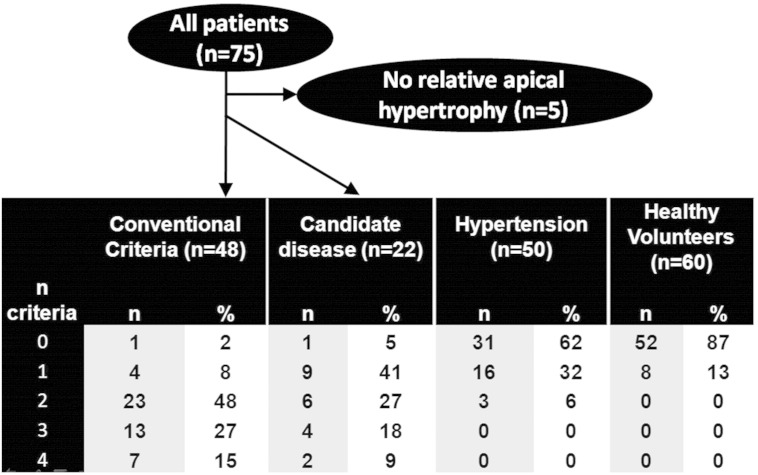
Patient flow diagram and table.

In the patients with ABR > 1, the incidence of 0,1,2,3,4 disease features was 5, 41, 27,18 and 9% respectively (Fig. 5). We suggest that the presence of 2 or more of these suggests an unequivocal disease phenotype (as in 54% of this cohort) whereas 0 or 1 is suspicious but may not be diagnostic.

## 8 Limitations

This preliminary dataset is not comprehensive — the individuals have not undergone: formal familial evaluation; conventional HCM risk assessment; comprehensive investigation of myocardial mechanics (regional function assessment, diastolic function, tagging); genotyping and there is no serial follow-up. Further limitations of this study include the minimal ethnic diversity in the hypertensive group, recruited from a single geographic area. The measurement of relative apical hypertrophy is difficult and was performed here in an experienced tertiary center specializing in CMR and cardiomyopathy. Therefore, the presence of additional imaging features is important. It was not possible to index the volume data since contemporaneous height and weights were not recorded. The hypertensive patients were consecutive and most were on ≤ 3 hypertensive agents and as such are a cohort of well controlled disease. Comparison with patients with severe hypertension would be informative.

## 9 Conclusion

We identify a group of patients with a distinct phenotype not captured by existing disease classifications. This phenotype comprises two essential criteria: ECG TWI, often deep, in the absence of other causes and relative apical hypertrophy (ABR > 1) detected by CMR. The presence of two or more of four minor criteria is supportive, the minor criteria being myocardial scarring by LGE CMR, the presence of apical aneurysm or microaneurysm, left atrial dilatation and apical cavity obliteration ≥ 20 mm. We postulate that this represents an extension of the apical HCM phenotype. Further work is required to define this entity and its prognostic significance.

## Conflict of interest

The authors report no relationships that could be construed as a conflict of interest

## Figures and Tables

**Table 1:** Patient and control subject characteristics.

	Disease candidate (n = 22)	HCM (n = 48)		Hypertension controls (n = 50)		Healthy controls (n = 60)	
			P		p		P
Age	50 ± 15	56 ± 11	0.10	55 ± 15	0.56	53 ± 17	0.78
Male gender n (%)	13 (60%)	41 (85%)	0.001	32 (64%)	0.56	35 (59%)	0.89
% White/Asian/Black	82/5/13	60/23/17	< 0.001	66/18/16	0.009	90/8/2	0.01
%TWI/deep TWI	27/73	21/79	n/a	0/0	n/a	0/0	n/a
EDV (ml)	128 ± 31	117 ± 24	0.09	144 ± 42	< 0.001	133 ± 31	0.007
ESV (ml)	31 ± 16	25 ± 10	0.06	47 ± 26	< 0.001	44 ± 14	< 0.001
SV (ml)	98 ± 21	92 ± 20	0.26	96 ± 24	0.32	89 ± 20	0.36
EF (%)	76 ± 8	79 ± 7	0.23	69 ± 11	< 0.001	68 ± 5	< 0.001
Mass (g)	156 ± 40	223 ± 69	< 0.001^a^	163 ± 64	0.01	123 ± 33	< 0.001
Left atrial area (cm^2^)	28 ± 7	27 ± 6	0.43	23 ± 4	< 0.001	20 ± 4	< 0.001
Max WT base (mm)	10 ± 1	14 ± 4	< 0.001^a^	11 ± 3	0.05	8 ± 1	< 0.001
Max WT apex (mm)	12 ± 1	17 ± 4	< 0.001^a^	6 ± 2	< 0.001	5 ± 1	< 0.001
Cavity oblit (mm)	23 ± 10	39 ± 15	< 0.001^a^	8 ± 5	< 0.001	4 ± 3	< 0.001
MAPSE (mm)	12 ± 3	11 ± 2	0.10	12 ± 3	0.10	13 ± 2	< 0.001
Number of antihypertensives. (0/1/2/3/4/5/6)				7/5/13/7/4/3/1			

a Unpaired T-Test vs disease candidate patients.

**Table 2:** Results.

	HCM (n = 48)	Disease candidate (n = 22)	Hypertensive controls (n = 50)	Healthy controls (n = 60)
Relative apical hypertrophy	36 (75%)	22 (100%)	0	0
Apex:base wall thickness ratio	1.2 ± 0.3	1.2 ± 0.1	0.5 ± 0.1	0.6 ± 0.1
LA dilated ≥ 25 mm	34 (71%)	15 (68%)	17 (34%)	8 (13%)
Apical obliteration				
> 10 mm	47 (98%)	21 (95%)	10 (20%)	0
> 20 mm	40 (83%)	14 (64%)	0	0
Apical microaneurysm	11 (23%)	3 (14%)	0	0
Scar	33 (67%)	9 (41%)	4 (8%)	0
