# Supplementary figures and images for: Diagnosis of apical hypertrophic cardiomyopathy: T-wave inversion and relative but not absolute apical left ventricular hypertrophy
Source: Int J Cardiol. 2015 Mar 15;183:143–8. doi: 10.1016/j.ijcard.2015.01.054 (PMC4392393; doi:10.1016/j.ijcard.2015.01.054)

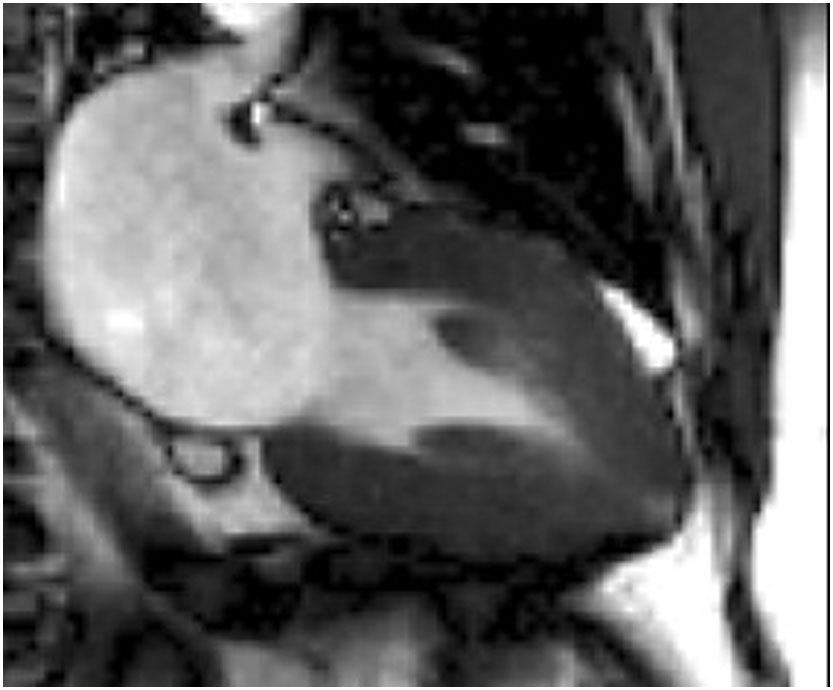

Supplement: Supplementary file 1 — Supplementary video 1. [file mmc1.jpg]

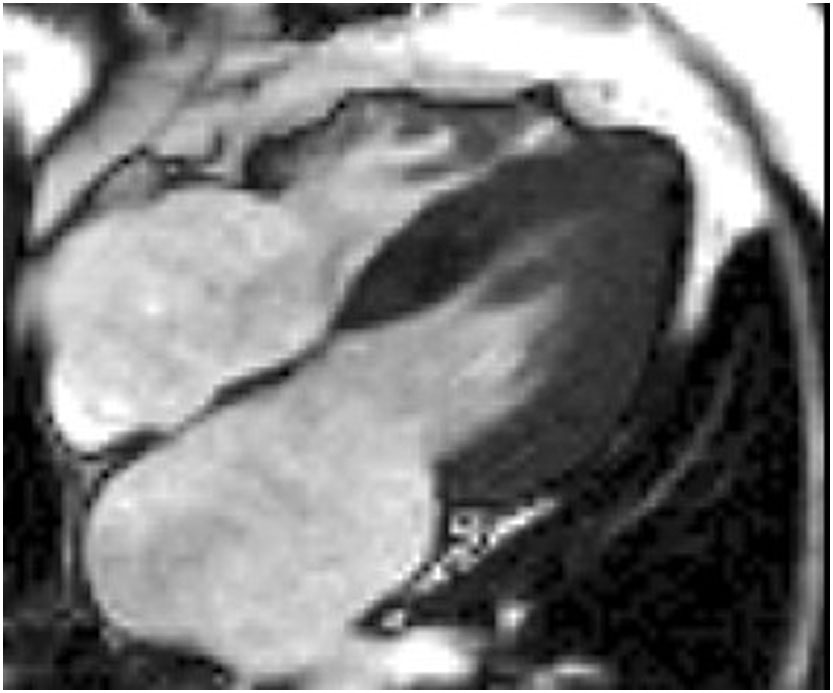

Supplement: Supplementary file 2 — Supplementary video 2. [file mmc2.jpg]
